# A214 AGE, SEX AND POSITION INFLUENCE UPPER ESOPHAGEAL SPHINCTER PRESSURES

**DOI:** 10.1093/jcag/gwad061.214

**Published:** 2024-02-14

**Authors:** K Pokraka, M Woo, D Randall

**Affiliations:** Alberta Health Services, Edmonton, AB, Canada; Alberta Health Services, Edmonton, AB, Canada; Alberta Health Services, Edmonton, AB, Canada

## Abstract

**Background:**

Upper esophageal sphincter (UES) function may be evaluated using high resolution manometry (HRM) to measure manometric parameters specific to the UES. The UES parameters are not well established however there is evidence suggesting a relationship between the UES parameters and esophageal motility disorders as well as UES parameters and symptoms including dysphagia, globus sensation and reflux. The Esophageal Symptom Questionnaire (ESQ-30) is a validated tool in the assessment of esophageal symptoms and measures the frequency and severity of dysphagia, reflux and globus through three separate sub-scales (ESQ-D, ESQ-R, ESQ-G respectively).

**Aims:**

We aimed to identify the relationship between UES manometric variables and patient demographic variables (age, sex) as well as patient symptoms including broad categories of globus sensation, dysphagia and reflux.

**Methods:**

Retrospective analysis of consecutive reports from HRM studies performed between October 2021 – March 2023 who provided consent for inclusion in the motility databank and completed ESQ-30 questionnaires. All values expressed as median (IQR: 25-75-centile). Values were compared with the non-parametric Mann-Whitney U test. Relationships between manometric diagnosis, UES pressures and symptomatology were explored.

**Results:**

681 patients underwent esophageal manometry and completed the ESQ-30 questionnaire. These patients were 442 (65%) female (mean age 54.6 +/- 14.8 years). Over the study period, 318 patients underwent esophageal HRM in the upright position and were diagnosed according to CCv3 and 361 patients underwent HRM in the supine position and were diagnosed by CCv4 manometric criteria.

UES mean basal pressure was significantly higher in females than males (64.6 [42.3-89.1] vs 68.4 [48.4-96.1], p = .015) as well as in the supine position (77.5 [55.9-101.0] vs. 56.0 [38.7-80.3]. UES mean basal pressure was negatively correlated with age (R^2^ -.28 p ampersand:003C .001).

In terms of esophageal symptomatology, dysphagia scores (ESQ-D) were negatively correlated with UES mean basal pressure (R^2^ -.15, p ampersand:003C .001).

**Conclusions:**

Age, sex and position may influence UES manometric pressures. The finding of higher UES pressures in the supine position may reflect a mechanism for decreasing proximal reflux. The decreased UES basal pressure with age is possibly related to changes in the integrity of the neuromuscular system. Having a better understanding of the poorly understood normal UES dynamics and how this changes in motility disorders may allow us to more specifically target areas for treatment.

**Funding Agencies:**

None

